# Determining exon connectivity in complex mRNAs by nanopore sequencing

**DOI:** 10.1186/s13059-015-0777-z

**Published:** 2015-09-30

**Authors:** Mohan T. Bolisetty, Gopinath Rajadinakaran, Brenton R. Graveley

**Affiliations:** Department of Genetics and Genome Sciences, Institute for Systems Genomics, University of Connecticut Health Center, Farmington, CT 06030 USA; Present Address: The Jackson Laboratory for Genomic Medicine, Farmington, CT 06030 USA

## Abstract

Short-read high-throughput RNA sequencing, though powerful, is limited in its ability to directly measure exon connectivity in mRNAs that contain multiple alternative exons located farther apart than the maximum read length. Here, we use the Oxford Nanopore MinION sequencer to identify 7,899 ‘full-length’ isoforms expressed from four *Drosophila* genes, *Dscam1*, *MRP*, *Mhc*, and *Rdl*. These results demonstrate that nanopore sequencing can be used to deconvolute individual isoforms and that it has the potential to be a powerful method for comprehensive transcriptome characterization.

## Background

High throughput RNA sequencing has revolutionized genomics and our understanding of the transcriptomes of many organisms. Most eukaryotic genes encode pre-mRNAs that are alternatively spliced [1]. In many genes, alternative splicing occurs at multiple places in the transcribed pre-mRNAs that are often located farther apart than the read lengths of most current high throughput sequencing platforms. As a result, several transcript assembly and quantitation software tools have been developed to address this [2, 3]. While these computational approaches do well with many transcripts, they generally have difficulty assembling transcripts of genes that express many isoforms. In fact, we have been unable to successfully assemble transcripts of complex alternatively spliced genes such as *Dscam1* or *Mhc* using any transcript assembly software (data not shown). These software tools also have difficulty quantitating transcripts that have many isoforms, and for genes with distantly located alternatively spliced regions, they can only infer, and not directly measure, which isoforms may have been present in the original RNA sample [4]. For example, consider a gene containing two alternatively spliced exons located 2 kbp away from one another in the mRNA. If each exon is observed to be included at a frequency of 50 % from short read sequence data, it is impossible to determine whether there are two equally abundant isoforms that each contain or lack both exons, or four equally abundant isoforms that contain both, neither, or only one or the other exon.

Pacific Bioscience sequencing can generate read lengths sufficient to sequence full length cDNA isoforms and several groups have recently reported the use of this approach to characterize the transcriptome [5]. However, the large capital expense of this platform can be a prohibitive barrier for some users. Thus, it remains difficult to accurately and directly determine the connectivity of exons within the same transcript. The MinION nanopore sequencer from Oxford Nanopore requires a small initial financial investment, can generate extremely long reads, and has the potential to revolutionize transcriptome characterization, as well as other areas of genomics.

Several eukaryotic genes can encode hundreds to thousands of isoforms. For example, in *Drosophila*, 47 genes encode over 1,000 isoforms each [6]. Of these, *Dscam1* is the most extensively alternatively spliced gene known and contains 115 exons, 95 of which are alternatively spliced and organized into four clusters [7]. The exon 4, 6, 9, and 17 clusters contain 12, 48, 33, and 2 exons, respectively. The exons within each cluster are spliced in a mutually exclusive manner and *Dscam1* therefore has the potential to generate 38,016 different mRNA and protein isoforms. The variable exon clusters are also located far from one another in the mRNA and the exons within each cluster are up to 80 % identical to one another at the nucleotide level. Together, these characteristics present numerous challenges to characterize exon connectivity within full-length *Dscam1* transcripts for any sequencing platform. Furthermore, though no other gene is as complex as *Dscam1*, many other genes have similar issues that confound the determination of exon connectivity.

We are interested in developing methods to perform simple and robust long-read sequencing of individual isoforms of *Dscam1* and other complex alternatively spliced genes. Here, we use the Oxford Nanopore MinION to sequence ‘full-length’ cDNAs from four *Drosophila* genes – *Rdl*, *MRP*, *Mhc*, and *Dscam1* – and identify a total of 7,899 distinct isoforms expressed by these four genes.

## Results and discussion

### Similarity between alternative exons

**Fig. 1:**
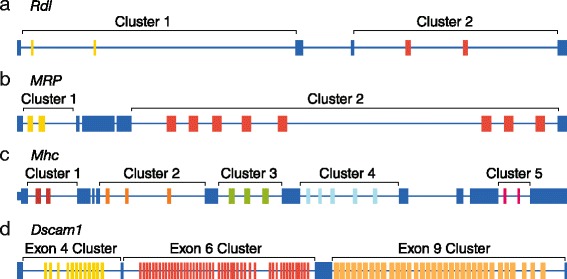
Schematic of the exon-intron structures of the genes examined in this study. **a** The *Rdl* gene contains two clusters (cluster one and two) which each contain two mutually exclusive exons. **b** The *MRP* gene contains contains two and eight mutually exclusive exons in clusters 1 and 2, respectively. **c** *Mhc* contains two mutually exclusive exons in clusters 1 and 5, three mutually exclusive exons in clusters 2 and 3, and five mutually exclusive exons in cluster 4. **d** The *Dscam1* gene contains 12, 48, and 33 mutually exclusive exons in the exon 4, 6, and 9 clusters, respectively. For each gene, the constitutive exons are colored blue, while the variable exons are colored yellow, red, orange, green, or light blue

We were interested in determining the feasibility of using the MinION nanopore sequencer to characterize the connectivity of distantly located exons in the mRNAs expressed from genes with complex splicing patterns. For the purposes of these experiments, we have focused on four *Drosophila* genes with increasingly complex patterns of alternative splicing (Fig. 1). *Resistant to dieldrin* (*Rdl*) contains two clusters, each containing two mutually exclusive exons and therefore has the potential to generate four different isoforms (Fig. 1a). *Multidrug-Resistance like Protein 1* (*MRP*) contains two mutually exclusive exons in cluster 1 and eight mutually exclusive exons in cluster 2, and can generate 16 possible isoforms (Fig. 1b). *Myosin heavy chain* (*Mhc*) can potentially generate 180 isoforms due to five clusters of mutually exclusive exons – clusters 1 and 5 contain two exons, clusters 2 and 3 each contain three exons, and cluster 4 contains five exons. Finally, *Dscam1* contains 12 exon 4 variants, 48 exon 6 variants, 33 exon 9 variants (Fig. 1d), and two exon 17 variants (not shown) and can potentially express 38,016 isoforms. For this study, however, we have focused only on the exon 3 through exon 10 region of *Dscam1*, which encompasses the 93 exon 4, 6, and 9 variants, and 19,008 potential isoforms (Fig. 1d).

**Fig. 2:**
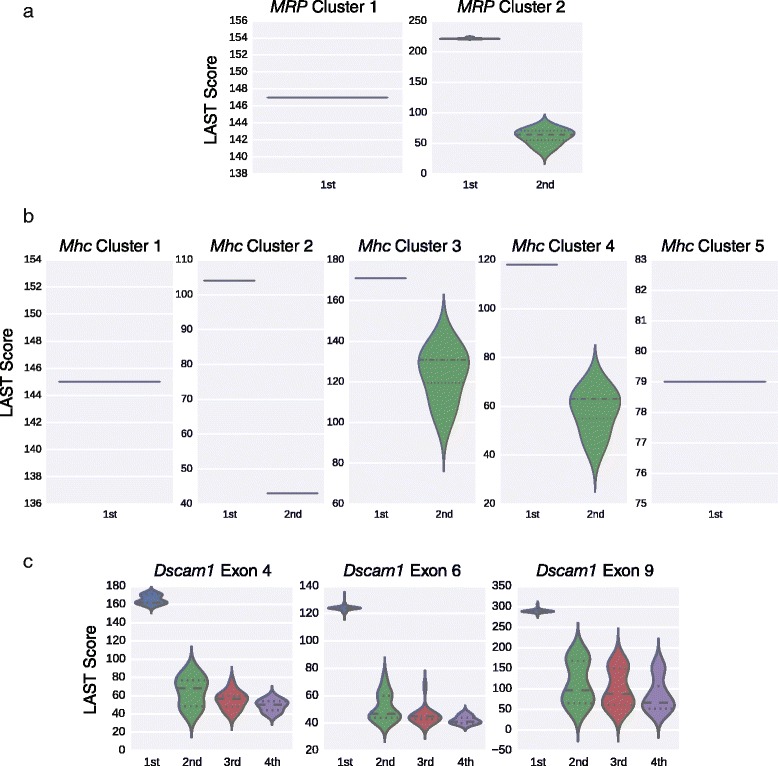
Similarity distance between the variable alternative exons of *MRP*, *Mhc*, and *Dscam1*. **a** Violin plots of the LAST alignment scores of each variable exon within *MRP* cluster 1 and MRP cluster 2 to themselves and the second (2nd) best alignments. **b** Violin plots of the LAST alignment scores of each variable exon within each *Mhc* cluster to themselves and the second (2nd) best alignments. **c** Violin plots of the LAST alignment scores of each variable exon within each *Dscam1* cluster to themselves (1st), and to the exons with the second (2nd), third (3rd) and fourth (4th) best alignments

Because our nanopore sequence analysis pipeline uses LAST to perform alignments [8], we aligned all of the *Rdl*, *MRP*, *Mhc*, and *Dscam1* exons within each cluster to one another using LAST to determine the extent of discrimination needed to accurately assign nanopore reads to a specific exon variant. For *Rdl*, each variable exon was only aligned to itself, and not to the other exon in the same cluster (data not shown). For *MRP*, the two exons within cluster 1 only align to themselves, and though the eight variable exons in cluster 2 do align to other exons, there is sufficient specificity to accurately assign nanopore reads to individual exons (Fig. 2a). For *Mhc*, the variable exons in cluster 1 and cluster 5 do not align to other exons, and the variable exons in cluster 2, cluster 3, and cluster 4 again align with sufficient discrimination to identify the precise exon present in the nanopore reads (Fig. 2b). Finally, for *Dscam1*, the difference in the LAST alignment scores between the best alignment (each exon to itself) and the second, third, and fourth best alignments are sufficient to identify the *Dscam1* exon variant (Fig. 2c). This analysis indicates that for each gene in this study, LAST alignment scores are sufficiently distinct to identify the variable exons present in each nanopore read.

### Optimizing template switching in *Dscam1* cDNA libraries

**Fig. 3:**
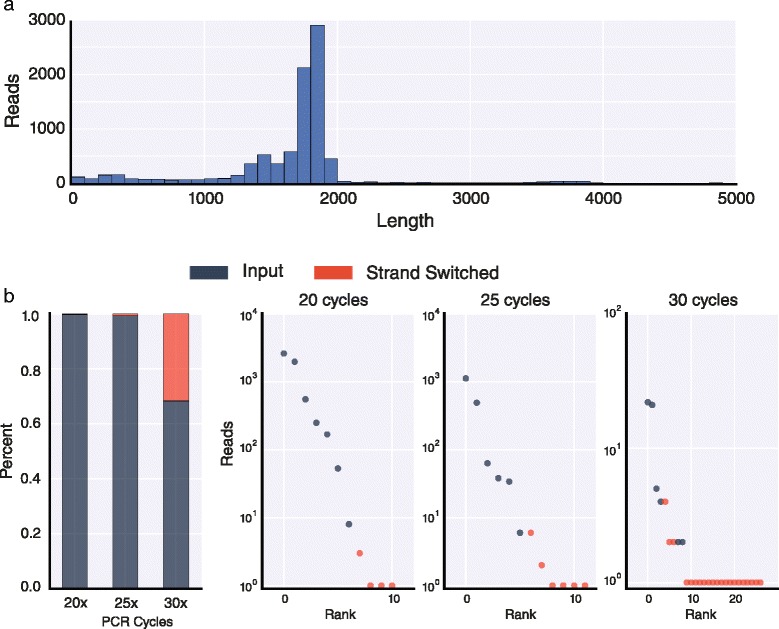
Optimized RT-PCR minimizes template-switching for MinION sequencing. **a** Histogram of read lengths from MinION sequencing of *Dscam1* spike-ins from the library generated using 25 cycles of PCR. **b** Bar plot indicating the extent of template switching in Dscam1 spike-ins at different PCR cycles (left). The blue portions indicate the fraction of reads corresponding to input isoforms while the red portions correspond to the fraction of reads corresponding to template-switched isoforms. On the right, plots of the rank order versus number of reads (log10) for the 20, 25, and 30 cycle libraries. The blue dots indicate input isoforms while the red portions correspond to template-switched isoforms

Template switching can occur frequently when libraries are prepared by PCR and can confound the interpretation of results [9, 10]. For example, CAM-Seq [11] and a similar method we independently developed called Triple-Read sequencing [12] to characterize *Dscam1* isoforms, were found to have excessive template switching due to amplification during the library prep protocols. To assess template switching in our current study, we generated a spike-in mixture of *in vitro* transcribed RNAs representing six unique *Dscam1* isoforms – *Dscam1*^*4.2,6.32,9.31*^, *Dscam1*^*4.1,6.46,9.30*^, *Dscam1*^*4.3,6.33,9.9*^, *Dscam1*^*4.12,6.44,9.32*^, *Dscam1*^*4.7,6.8,9.15*^, and *Dscam1*^*4.5,6.4,9.4*^. We used 10 pg of this control spike-in mixture and prepared libraries for MinION sequencing by amplifying the exon 3 through exon 10 region for 20, 25, or 30 cycles of RT-PCR. We then end-repaired and dA-tailed the fragments, ligated adapters, and sequenced the samples on a MinION (7.3) for 12 h each. We obtained 33,736, 8,961, and 7,511 base-called reads from the 20, 25, and 30 cycle libraries, respectively. Consistent with the size of the exon 3 to 10 cDNA fragment being 1,806–1,860 bp in length, depending on the precise combination of exons it contains, most reads we observed were in this size range (Fig. 3a). We used Poretools [13] to convert the raw output files into fasta format and then used LAST to align the reads to a LAST database containing each variable exon. From these alignments, we identified reads that mapped to all three exon clusters, as well as the exon with the best alignment score within each cluster. When examining the alignments to each cluster independently, we found that for these spike-in libraries, all reads mapped uniquely to the exons present in the input isoforms. Therefore, any observed isoforms that were not present in the input pool were a result of template switching during the RT-PCR and library prep protocol and not due to false alignments or sequencing errors.

When comparing the combinations of exons within each read to the input isoforms, we observed that 32 % of the reads from the 30 cycle library corresponded to isoforms generated by template switching (Fig. 3b). The template-switched isoforms observed by the greatest number of reads in the 30 cycle library were due to template switching between the two most frequently sequenced input isoforms. In most cases, template switching occurred somewhere within exon 7 or 8 and resulted in a change in exon 9. However, the extent of template switching was reduced to only 1 % in the libraries prepared using 25 cycles, and to 0.2 % in the libraries prepared using 20 cycles of PCR (Fig. 3b). Again, for these two libraries the most frequently sequenced template-switched isoforms involved the input isoforms that were also the most frequently sequenced. These experiments demonstrate that the MinION nanopore sequencer can be used to sequence ‘full length’ *Dscam1* cDNAs with sufficient accuracy to identify isoforms and that the cDNA libraries can be prepared in a manner that results in a very small amount of template switching.

### *Dscam1* isoforms observed in adult heads

**Fig. 4:**
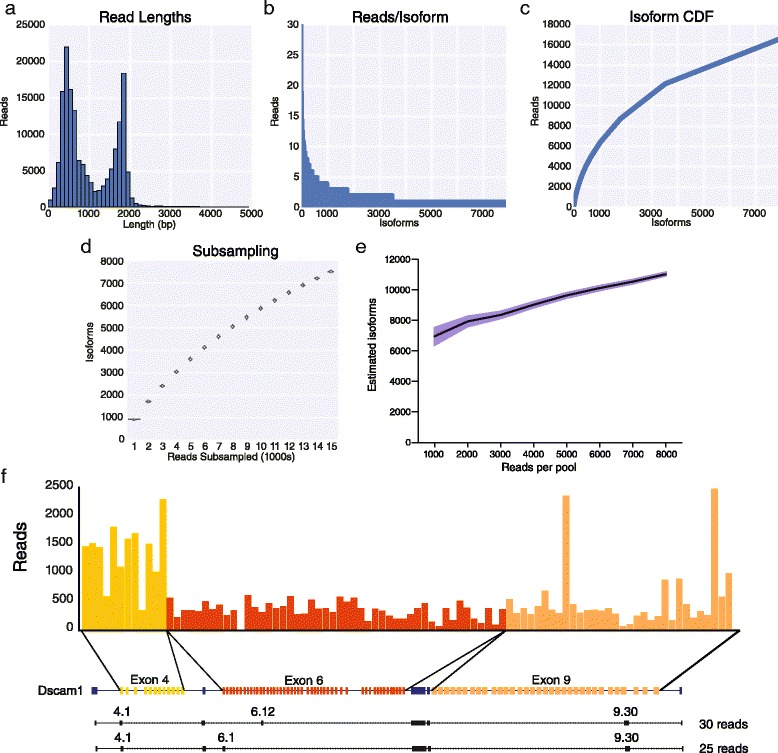
MinION sequencing of *Dscam1* identified 7,874 isoforms. **a** Histogram of read length distribution for *Drosophila* head samples. **b** The total number of *Dscam1* isoforms identified from MinION sequencing. **c** Cumulative distribution of *Dscam1* isoforms with respect to expression. **d** Violin plot of the number of isoforms identified using 100 random pools of the indicated number of reads. **e** Plot of the estimated number of total isoforms present in the library using the capture-recapture method with two random pools of the indicated number of reads. The shaded blue area indicates the 95 % confidence interval. **f** Deconvoluted expression of *Dscam1* exon cluster variants (top) and the isoform connectivity of two highly expressed *Dscam1* isoforms (bottom)

**Fig. 5:**
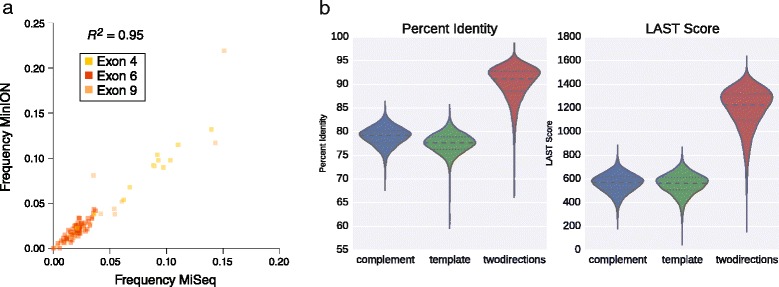
Accuracy of *Dscam1* sequencing results. **a** Comparison of the frequency of variable exon inclusion for the *Dscam1* exon 4 (yellow), 6 (red), and 9 (orange) clusters as determined by nanopore sequencing or by amplicon sequencing using an Illumina MiSeq. **b** Percent identities (left) or LAST alignment scores (right) of full-length template, complement, and two directions (sequencing both template and complements) nanopore read alignments

To explore the diversity of *Dscam1* isoforms expressed in a biological sample, we prepared a *Dscam1* library from RNA isolated from *D. melanogaster* heads prepared from mixed male and female adults using 25 cycles of PCR and sequenced it for 12 h on the MinION nanopore sequencer obtaining a total of 159,948 reads of which 78,097 were template reads, 48,474 were complement reads, and 33,377 were 2D reads (Fig. 4a). We aligned the reads individually to the exon 4, 6, and 9 variants using LAST. A total of 28,971 reads could be uniquely or preferentially aligned to a single variant in all three clusters. For further analysis, we used all 16,419 2D read alignments and 31 1D reads when both template and complement aligned to same variant exons (not all reads with both a template and complement yield a 2D read). The remaining 12,521 aligned reads were 1D reads where there was either only a template or complement read, or when the template and complement reads disagreed with one another and were therefore not used further. We observed 92 of the 93 potential exon 4, 6, or 9 variants – only exon 6.11 was not observed in any read (Fig. 4f). To assess the accuracy of the results we performed RT-PCR using primers in the flanking constitutive exons that contained Illumina sequencing primers to separately amplify the *Dscam1* exon 4, 6, and 9 clusters from the same RNA used to prepare the MinION libraries, and sequenced the amplicons on an Illumina MiSeq. The frequency of variable exon use in each cluster was extremely consistent between the two methods (*R*^*2*^ = 0.95, Fig. 5a).

Over their entire lengths, the 2D reads that map specifically to one exon 4, 6, and 9 variants map with an average 90.37 % identity and an average LAST score of approximately 1,200 (Fig. 5b). The 16,450 full length reads correspond to 7,874 unique isoforms, or 42 % of the 18,612 possible isoforms given the exon 4, 6, and 9 variants observed. We note, however, that while 4,385 isoforms were represented by more than one read, 3,516 of isoforms were represented by only one read indicating that the depth of sequencing has not reached saturation (Fig. 4b and c). This was further confirmed by performing a bootstrapped subsampling analysis (Fig. 4d) and by using the capture-recapture method to attempt to assess the complexity of isoforms present in the library (Fig. 4e), which suggests that over 11,000 isoforms are likely to be present, though even this analysis has not yet reached saturation. The most frequently observed isoforms were *Dscam1*^*4.1,6.12,9.30*^ and *Dscam1*^*4.1,6.1,9.30*^ which were observed with 30 and 25 reads, respectively (Fig. 4e). In conclusion, these results demonstrate the practical application of using the MinION nanopore sequencer to identify thousands of distinct *Dscam1* isoforms in a single biological sample.

### Nanopore sequencing of ‘full-length’ *Rdl*, *MRP*, and *Mhc* isoforms

**Fig. 6:**
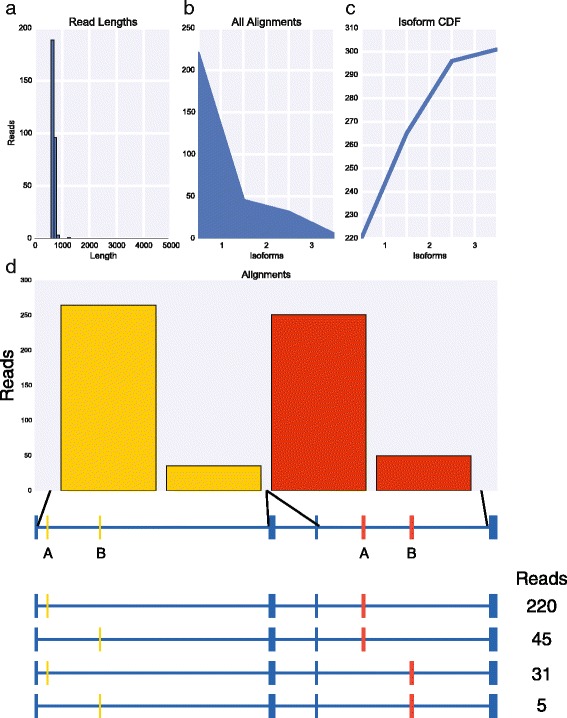
MinION sequencing of *Rdl* identified four isoforms. **a** Histogram of read lengths. **b** The number of reads per isoform. **c** Cumulative distribution of isoforms with respect to expression. **d** The number of reads per alternative exon (top) and per isoform (below)

**Fig. 7:**
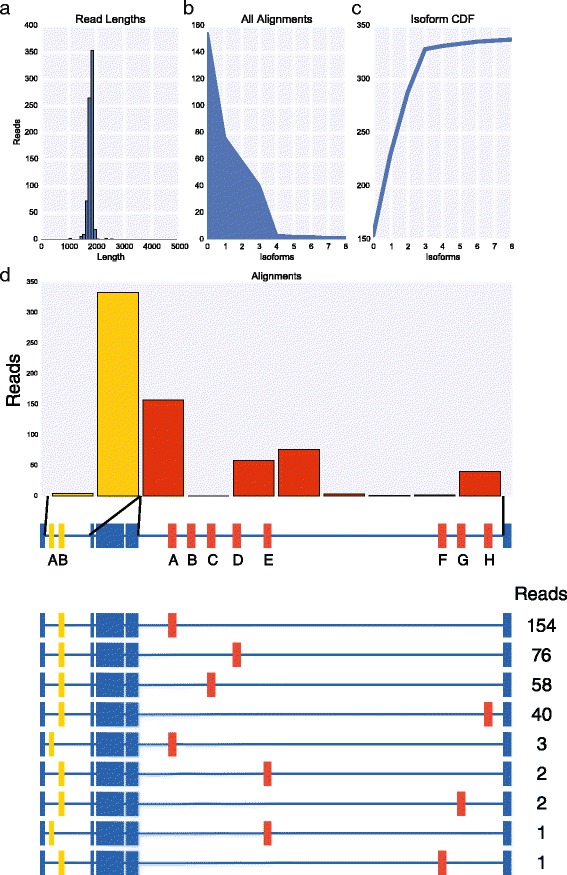
MinION sequencing of *MRP* identified nine isoforms. **a** Histogram of read lengths. **b** The number of reads per isoform. **c** Cumulative distribution of isoforms with respect to expression. **d** The number of reads per alternative exon (top) and per isoform (below)

**Fig. 8:**
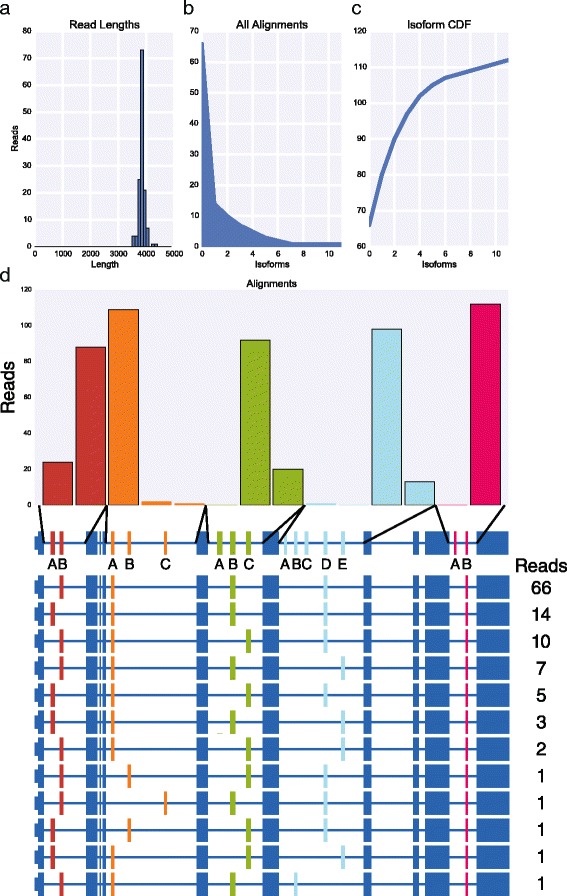
MinION sequencing of *Mhc* identified 12 isoforms. **a** Histogram of read lengths. **b** The number of reads per isoform. **c** Cumulative distribution of isoforms with respect to expression. **d** The number of reads per alternative exon (top) and per isoform (below)

To extend this approach to other genes with complex splicing patterns, we focused on *Rdl*, *MRP*, and *Mhc* which have the potential to generate four, 16, and 180 isoforms, respectively. We prepared libraries for each of these genes by RT-PCR using primers in the constitutive exons flanking the most distal alternative exons using 25 cycles of PCR, pooled the three libraries and sequenced them together on the MinION nanopore sequencer for 12 h obtaining a total of 22,962 reads. The input libraries for *Rdl*, *MRP*, and *Mhc* were 567 bp, 1,769-1,772 bp, and 3,824 bp, respectively. The raw reads were aligned independently to LAST indexes of each cluster of variable exons. The alignment results were then used to assign reads to their respective libraries, identify reads that mapped to all variable exon clusters for each gene, and the exon with the best alignment score within each cluster. In total, we obtained 301, 337, and 112 full length reads for *Rdl* (Fig. 6), *MRP* (Fig. 7), and *Mhc* (Fig. 8), respectively. For *Rdl*, both variable exons in each cluster was observed, and accordingly all four possible isoforms were observed, though in each case the first exon was observed at a much higher frequency than the second exon (Fig. 6d). Interestingly, the ratio of isoforms containing the first versus second exon in the second cluster is similar for isoforms containing either the first exon or the second exon in the first cluster indicating that the splicing of these two clusters may be independent. For *MRP*, both exons in the first cluster were observed and all but one of the exons in the second cluster (exon B) were observed, though the frequency at which the exons in both clusters were used varied dramatically (Fig. 7d). For example, within the first cluster, exon B was observed 333 times while exon A was observed only four times. Similarly, in the second cluster, exon A was observed 157 times whereas exons B, E, F, and G were observed 0 times, thrice, once, and twice, respectively, and exons D, E, and H were observed between 40 and 76 times. As a result, we observed only nine *MRP* isoforms. For *Mhc*, we again observed strong biases in the exons observed in each of the five clusters (Fig. 8d). In the first cluster, exon B was observed more frequently than exon A. In the second cluster, 109 of the reads corresponded to exon A, while exons B and C were observed by only two and one read, respectively. In the third cluster, exon A was not observed at all while exons B and C were observed in roughly 80 % and 20 % of reads, respectively. In the fourth cluster, exon A was observed only once, exons B and C were not observed at all, exon E was observed 13 times while exon D was present in all of the remaining reads. Finally, in the fifth cluster, only exon B was observed. As with *MRP*, these strong biases and near or complete absences of exons in some of the clusters severely reduces the number of possible isoforms that can be observed. In fact, of the 180 potential isoforms encoded by *Mhc*, we observed only 12 isoforms. Various *Mhc* isoforms are known to be expressed in striking spatial and temporally restricted patterns [14] and thus it is likely that other *Mhc* isoforms that we did not observe, could be observed by sequencing other tissue samples.

## Conclusions

Here we have demonstrated that nanopore sequencing with the Oxford Nanopore MinION can be used to easily determine the connectivity of exons in a single transcript, including *Dscam1*, the most complicated alternatively spliced gene known in nature. This is an important advance for several reasons. First, because short-read sequence data cannot be used to conclusively determine which exons are present in the same RNA molecule, especially for complex alternatively spliced genes, long-read sequence data are necessary to fully characterize the transcript structure and exon connectivity of eukaryotic transcriptomes. Second, although the Pacific Bioscience platform can perform long-read sequencing, there are several differences between it and the Oxford Nanopore MinION that could cause users to choose one platform over the other. In general, the quality of the sequence generated by the Pacific Bioscience is higher than that currently generated by the Oxford Nanopore MinION. This is largely due to the fact that each molecule is sequenced multiple times on the Pacific Bioscience platform yielding a high quality consensus sequence whereas on the Oxford Nanopore MinION, each molecule is sequenced at most twice (in the template and complement). We have previously used the Pacific Bioscience platform to characterize *Dscam1* isoforms and found that it works well, though due to the large amount of cDNA needed to generate the libraries, many cycles of PCR are necessary and we observed an extensive amount of template switching, making it impractical to use for these experiments (BRG, unpublished data). However, over the past year that we have been involved in the MAP, the quality of sequence has steadily increased. As this trend is likely to continue, the difference in sequence quality between these two platforms is almost certain to shrink. Nonetheless, as we demonstrate, the current quality of the data is more than sufficient to allow us to accurately distinguish between highly similar alternatively spliced isoforms of the most complex gene in nature. Third, the ability to accurately characterize alternatively spliced transcripts with the Oxford Nanopore MinION makes this technology accessible to a much broader range of researchers than was previously possible. This is in part due to the fact that, in contrast to all other sequencing platforms, very little capital expense is needed to acquire the sequencer. Moreover, the MinION is truly a portable sequencer that could literally be used in the field (provided one has access to an Internet connection), and due to its size, almost no laboratory space is required for its use.

Although nanopore sequencing has many exciting and potentially disruptive advantages, there are several areas in which improvement is needed. First, although we were able to accurately identify over 7,000 *Dscam1* isoforms with an average identity of full-length alignments >90 %, there are several situations in which this level of accuracy will be insufficient to determine transcript structure. For instance, there are many micro-exons in the human genome [15], and these exons would be difficult to identify if they overlapped a portion of a read that contained errors. Additionally, small unannotated exons could be difficult to identify for similar reasons. Second, the current number of usable reads is lower than that which will be required to perform whole transcriptome analysis. One issue that plagues transcriptome studies is that the majority of the sequence generated comes from the most abundant transcripts. Thus, with the current throughput, numerous runs would be needed to generate a sufficient number of reads necessary to sample transcripts expressed at a low level. In fact, this is one reason that we chose in this study, to begin by targeting specific genes rather than attempting to sequence the entire transcriptome. We do note, however, that over the past year of our participation in the MAP, the throughput of the Oxford Nanopore MinION has increased, and it is reasonable to expect additional improvements in throughput that should make it possible to generate a sufficient number of long reads to deeply interrogate even the most complex transcriptome.

In conclusion, we anticipate that nanopore sequencing of whole transcriptomes, rather than targeted genes as we have performed here, will be a rapid and powerful approach for characterizing isoforms, especially with improvements in the throughput and accuracy of the technology, and the simplification and/or elimination of the time-consuming library preparations.

## Materials and methods

### *Drosophila* strains

*Drosophila melanogaster y*; *cn b sp* (stock: 2057, Bloomington) were maintained and raised at room temperature.

### Spike-in preparation

Total RNA from about 30 heads was extracted using Trizol reagent. One microgram of total RNA was used to synthesize cDNA using random hexamers with SuperScript II (Invitrogen, Cat No: 18064) in a 20 μL reaction; 2 μl of cDNA reaction was used to amplify *Dscam1* exons 4 through 9 using the primers exon 3 and exon 10 with LongAmp (New England Biolabs, Cat No: M0323) in a 50 μL reaction volume with the following PCR condition: initial denaturation at 94 °C for 30 s, denaturation at 94 °C for 15 s, annealing at 58 °C for 15 s, extension at 65 °C for 100 s (40X cycle), final extension at 65 °C for 10 min. The PCR amplicons were purified using MinElute PCR purification kit (Qiagen) and eluted in 20 μL ultrapure water. The eluted amplicons were then cloned into a vector with both T7 and SP6 dual promoters (Life Technologies, Cat No: K4600) and transformed into Top10 shot cells. A total of 96 colonies were sequenced to identify exon variant sequences in individual clones. Six individual colonies containing a single, non-overlapping, unique exon variants were used to make spike-in RNAs. The vector containing the *Dscam1* insert and the T7, SP6 promoter sequences were amplified using M13F and M13R primers. The SP6 oriented clones were individually amplified using T7 overhang primers to facilitate *in vitro* transcription of all clones from T7 promoter using transcription kit. Following transcription, 1 μL RNA (1 μg/μL) of each of the six clones were mixed and a 10-fold serial dilution was made with concentration ranging from 100 ng/μL to 1 pg/μL. cDNA was synthesized using SuperScript II (Invitrogen, Cat No: 18064) and a 2.5 μL cDNA from 10 pg/μL reaction was used in the 25 μL Phusion PCR with the following conditions: initial denaturation at 95 °C for 30 s, denaturation at 95 °C for 10 s, annealing at 64.7 °C for 12 s, extension at 72 °C for 40 s (20X, 25X, and 30X cycles), final extension at 72 °C for 5 min, using primers CGGATCCATTATCTCCCGGGACG (*Dscam1* exon 3) and CGGATCCCTGGGCGAAGGCC (*Dscam1* exon 10 reverse).

### Amplicon library preparation and Oxford Nanopore sequencing

The library preparation for amplicon sequencing was done using SQK-MAP003 following manufacturer’s protocol (ONT). Briefly, a total of 850 ng (spike-in) and 1 μg (mixed heads) in 80 μL was end repaired using NEBNext End Repair Module (New England Biolabs, Cat No: E6050) and followed by dA tailing using NEBNext dA Tailing Module (New England Biolabs, Cat No: E6053). The dA tailed amplicons were then adapter ligated in a total of 100 μL reaction volume and incubated at room temperature for 10 min. This reaction mixture was then purified using Agencourt AMPure XP (Beckman Coulter Inc., cat. no. A63880) beads and washed and eluted in nanopore supplied reagents in 25 μL ultrapure water. This pre-sequencing mix was added with the fuel mix and EP buffer and loaded on the R7.3 flow cell and sequenced.

### Nanopore data analysis

Poretools (version 0.3.0) [13] was used to extract fasta reads from Basecalled fast5 files. Exon cluster specific LAST indices were made using lastdb with default parameters. The reads were then aligned using lastal independently to these LAST indices using the following parameters: −s 2 -T 0 -Q 0 -a 1. Reads that aligned to all three clusters were parsed from all alignments and used for further processing. The top scoring alignment was used for reads that aligned to multiple variants. iPython notebooks containing all the analysis and code are available at github/mohanbolisetty/dscam_nanopore. MAF files from LAST alignments were converted to SAM or PSL formats using maf-convert.py.

### *Dscam1* variable exon amplicon library preparation and Illumina sequencing

For the *Dscam1* MiSeq amplicon library, cDNA was synthesized using 1 μL RNA (600 ng/μL) from mixed heads using SuperScript II (Invitrogen, Cat No: 18064) in a 20 μL reaction. A total of 2.5 μL of cDNA was used to individually amplify the exon 4, 6, and 9 clusters with Phusion (NEB Inc., catalog no. M0530L) using the following PCR protocol: 95 °C for 30 s followed by 30 cycles of 95 °C for 10 s, 59 °C for 12 s and 72 °C for 15 s, followed by a 5 min incubation at 72 °C using the following primer pairs:

- Cluster4_Fwd: AATGATACGGCGACCACCGAGATCTACACCTCTCTATACACTCTTTCCCTACACGACGCTCTTCCGATCTATCggcaataccaggtactttcc
- Cluster4_Rev: CAAGCAGAAGACGGCATACGAGATCTAGTACGGTGACTGGAGTTCAGACGTGTGCTCTTCCGATCTATCgatccattatctcccggga
- Cluster6_Fwd: AATGATACGGCGACCACCGAGATCTACACACTGCATAACACTCTTTCCCTACACGACGCTCTTCCGATCTATCtgttccttcgatgaacttgt
- Cluster6_Rev: CAAGCAGAAGACGGCATACGAGATCATGCCTAGTGACTGGAGTTCAGACGTGTGCTCTTCCGATCTATCttaagtgccacaaaaggacg
- Cluster9_Fwd: AATGATACGGCGACCACCGAGATCTACACACCTCTTCACACTCTTTCCCTACACGACGCTCTTCCGATCTTCctcgaggatccatctggg
- Cluster9_Rev: CAAGCAGAAGACGGCATACGAGATTGCCTCTTGTGACTGGAGTTCAGACGTGTGCTCTTCCGATCTTCtcgaggatctctggaagtg

Following amplification, three separate PCR reactions were mixed together and purified using Agencourt AMPure XP (Beckman Coulter Inc., cat. no. A63880) beads. A library concentration of 2.1 nM was loaded and sequenced using MiSeq® Reagent Kit v3 (Illumina Inc., cat no. MS-102-3001).

### MiSeq data analysis

The fastq files were processed in R using the package Biostrings [16]. The reverse primer sequences from each of the *Dscam1* exon 4, 6, and 9 clusters were matched (allowing no mismatches) against fasta sequences from read 2. The matching reads were subsequently aligned against each reference exon variant (length trimmed to 51 bp from the start of each variant) within a cluster for all three clusters.

### Accession number

The raw nanopore data are available at the European Nucleotide Archive (ENA) under accession number ERP011508.

## References

[1] Nilsen TW, Graveley BR (2010). Expansion of the eukaryotic proteome by alternative splicing. Nature.

[2] Trapnell C, Williams BA, Pertea G, Mortazavi A, Kwan G, van Baren MJ (2010). Transcript assembly and quantification by RNA-Seq reveals unannotated transcripts and isoform switching during cell differentiation. Nat Biotechnol.

[3] Grabherr MG, Haas BJ, Yassour M, Levin JZ, Thompson DA, Amit I (2011). Full-length transcriptome assembly from RNA-Seq data without a reference genome. Nat Biotechnol.

[4] Garber M, Grabherr MG, Guttman M, Trapnell C (2011). Computational methods for transcriptome annotation and quantification using RNA-seq. Nat Methods.

[5] Sharon D, Tilgner H, Grubert F, Snyder M (2013). A single-molecule long-read survey of the human transcriptome. Nat Biotechnol.

[6] Brown JB, Boley N, Eisman R, May GE, Stoiber M, Booth BW (2014). Diversity and dynamics of the Drosophila transcriptome. Nature.

[7] Schmucker D, Clemens JC, Shu H, Worby CA, Xiao J, Muda M (2000). Drosophila Dscam is an axon guidance receptor exhibiting extraordinary molecular diversity. Cell.

[8] Frith MC, Hamada M, Horton P (2010). Parameters for accurate genome alignment. BMC Bioinformatics.

[9] McManus CJ, Duff MO, Eipper-Mains J, Graveley BR (2010). Global analysis of trans-splicing in Drosophila. Proc Natl Acad Sci U S A.

[10] Plocik AM, Graveley BR (2013). New insights from existing sequence data: generating breakthroughs without a pipette. Mol Cell.

[11] Sun W, You X, Gogol-Doring A, He H, Kise Y, Sohn M (2013). Ultra-deep profiling of alternatively spliced Drosophila Dscam isoforms by circularization-assisted multi-segment sequencing. EMBO J.

[12] Roy CK, Olson S, Graveley BR, Zamore PD, Moore MJ. Assessing long-distance RNA sequence connectivity via RNA-templated DNA-DNA ligation. Elife 2015, 4. 10.7554/eLife.03700 PMC4442144 25866926

[13] Loman NJ, Quinlan AR (2014). Poretools: a toolkit for analyzing nanopore sequence data. Bioinformatics.

[14] Zhang X, Bernstein SI (2001). Spatially and temporally regulated expression of myosin heavy chain alterantive exons during Drosophila embryogenesis. Mech Dev.

[15] Irimia M, Weatheritt RJ, Ellis JD, Parikshak NN, Gonatopoulos-Pournatzis T, Babor M (2014). A highly conserved program of neuronal microexons is misregulated in autistic brains. Cell.

[16] Pages H, Aboyoun P, Gentleman R, DebRoy S. Biostrings: String objects representing biological sequences, and matching algorithms, R package version 2.34.1 edition.

